# Comparative Pathology of Pseudorabies in Different Naturally and Experimentally Infected Species—A Review

**DOI:** 10.3390/pathogens9080633

**Published:** 2020-08-04

**Authors:** Julia Sehl, Jens Peter Teifke

**Affiliations:** Department of Experimental Facilities and Biorisk Management, Friedrich-Loeffler-Institut, 17493 Greifswald-Insel Riems, Germany; JensPeter.Teifke@fli.de

**Keywords:** pseudorabies virus, Aujeszky’s disease, clinical signs, necropsy, histopathology, porcine, non-porcine species, natural infection, experimental infection

## Abstract

The pseudorabies virus (PRV) is an alphaherpesvirus and the causative agent of Aujeszky’s disease (AD). PRV infects a wide range of animal species including swine as the natural host as well as ruminants, carnivores, rodents and lagomorphs. In these species, except for the pig, PRV infection causes acute, severe disease, characterized by insatiable itching, and is always lethal. Horses, chickens and non-human primates have been shown to be largely resistant to PRV infection, while disease in humans is still controversial. PRV is a pantropic virus, which preferably invades neural tissue, but also infects epithelia of various organs, whereupon multisystemic lesions may result. Although AD is mainly associated with severe pruritus, also known as “mad itch”, there are notable differences regarding infection route, clinical signs, viral distribution and lesion patterns in different animal species. In this comprehensive review, we will present clinico-pathologic findings from different species, which have been either shown to be susceptible to PRV infection or have been tested experimentally.

## 1. Introduction

Pseudorabies, also known as Aujeszky’s disease (AD) or mad itch, is caused by pseudorabies virus (PRV), syn. Aujeszky’s disease virus (ADV), or *Suid alphaherpesvirus* 1 (SuHV-1). Although the taxonomic species name may imply that the only natural reservoir and host species for PRV is pigs [1], the disease was first recorded in 1813 in cattle in the United States [2]. The resemblance of the clinical picture in ruminants to rabies led to the term “pseudorabies” in Switzerland in 1849 [3]. Likewise, “mad itch” was used as a descriptor for the clinical course in cattle in the United States throughout the first half of the 19th century. For the first time, the cause was described in 1902 by the veterinary pathologist and microbiologist Aladár Aujeszky in Hungary. He isolated the infectious agent from the brains of a diseased ox, a dog, and a cat, and differentiated AD from rabies as the main clinical differential diagnosis [4]. Shope demonstrated that the filterable virus, which caused “mad itch” in cattle in the midwestern United States, was immunologically identical to the Hungarian (Aujeszky’s) strain of PRV and showed its presence in domestic pig holdings in the United States [5]. Erich Traub in Germany cultivated PRV in vitro [6]. The serological relation between PRV and herpes simplex virus was discovered by Sabin and Wright (1934) [7], leading to the inclusion of PRV into the herpesvirus family.

Today, it is known that PRV can infect numerous other mammals, including mainly ruminants, carnivores and rodents. In all affected animal species other than the pig, the course of disease is invariably fatal and characterized by excessive pruritus and severe central nervous signs with a mortality of nearly 100% [8].

The present review focuses on the clinical picture as well as on post-mortem findings including gross and microscopic pathology in different animal species susceptible to PRV infection. We will highlight the special features of AD in the pig as the natural host as well as in non-porcine species including domestic and wild carnivores, ruminants, horses, chickens, laboratory animals and men.

## 2. Etiology

PRV is a DNA virus and is classified into the genus *Varicellovirus*, subfamily *Alphaherpesvirinae*, family *Herpesviridae* [9]. Only one serotype of this neurotropic herpesvirus exists with four major genotypes I–IV. Type I is found predominantly in the United States and central Europe, whereas types II and III circulate in central and northern Europe, respectively. Type IV is restricted to Asia [10]. The full genome sequence of PRV was elucidated in 2004 [11], and further full genome sequences from strains isolated in different countries are now available [12,13]. The PRV genome encompasses approximately 140 kbp and contains at least 72 open reading frames (ORFs) encoding 70 different proteins [11].

## 3. Epidemiology

The substantial economic losses to the swine industry are the reason that AD is an important notifiable and World Organization for Animal Health (O.I.E.) listed disease. Since the early 1980s, AD had spread almost globally, mainly as sequela of international movement of animals and animal products [14]. Due to strict animal disease control measures and eradication programs, including campaigns with gE-deleted differentiating infected from vaccinated animals (DIVA)-vaccines, AD has virtually disappeared from domestic pigs in several parts of Europe, e.g., Austria, Cyprus, the Czech Republic, Denmark, Finland, France (except a few departments), Germany, Hungary, Luxembourg, the Netherlands, Sweden, Switzerland, Slovakia, Norway, and Great Britain. More recently, Canada, New Zealand and the United States have also become free of AD in domestic pigs [14]. Today, AD is still endemic in areas with dense pig populations, i.e., some regions in eastern and southeastern Europe, Latin America, Africa, and Asia. The role of wild boar as potential and persistent reservoirs for PRV has become increasingly obvious and important [14,15], since infected wild boar represent a threat for reintroduction of PRV into free regions.

## 4. Aujeszky’s Disease in Mammals

Pigs are the only natural host for PRV in which latent infection may occur, but the virus can naturally infect cattle, sheep, cats, dogs, rabbits, mice, rats and guinea pigs causing fatal disease [16]. It is generally accepted that humans are highly resistant against natural PRV infection and even self-inoculation does not provoke disease [17]. However, recently isolated cases of PRV infection have been reported in humans who had close contact to swine or pork [18]. There are numerous reports of PRV infection in dogs, especially associated with hunting [14,19,20]. Pseudorabies has also been reported in wildlife, like wild boar, brown bear, black bear, Florida panther, raccoon, coyote, and farm fur animal species like mink and foxes [21,22,23]. Referring to laboratory animals, the rabbit is the most susceptible species. Besides its relevance as an animal pathogen, PRV has become more and more attractive as a model for the study of herpesviral pathogenesis at the molecular level. Genetically engineered viral variants expressing specific reporter proteins can be used to track virus infection in experimental animal models, of which laboratory mice and rats play an important role [24,25,26,27].

In the following, the course of AD including gross and microscopic pathology is summarized for different animal species. A detailed description of clinical appearance, gross and histopathological findings after natural and experimental infection is given in Supplementary Table S1.

### 4.1. AD in Swine

#### 4.1.1. General Information

Swine including domestic pigs and wild boar represent the natural host of PRV where the virus causes neurological, respiratory and reproductive disease. Infected pigs shed large quantities of virus in secretions and excretions. Thus, PRV is mainly spread via direct contact, but may also be transmitted by air, water and contaminated fomites [18]. PRV can be transmitted between pigs orally, nasally, transplacentally, and venereally. The clinical outcome is strongly dependent on the age of the infected animal [28]. Morbidity and mortality rates are higher in suckling and weaning pigs, and the disease is primarily associated with neurological signs. Respiratory or reproductive disorders are rather observed in adult pigs [29,30,31,32,33,34]. Besides reports on natural infection in both wild boar and domestic pigs [28,35,36,37,38], studies have been conducted to investigate AD in pigs experimentally [15,34,39,40,41,42,43,44,45,46,47,48]. It has been shown that pigs are susceptible to the intramuscular (i.m.), subcutaneous (s.c.) and peroral (p.o.) route of infection, but intranasal (i.n.) inoculation has been proven to be very efficient to induce AD experimentally.

#### 4.1.2. Pathogenesis

PRV first replicates in epithelial cells of the upper respiratory tract, from where it gains access to lymph and blood vessels, and is then transported to the tonsils and tributary lymph nodes. Following cell-free or cell-associated viremia mainly in monocytes, PRV infects a multitude of organs and is able to replicate in endothelial cells, epithelial cells of various tissues and in lymphocytes and macrophages [49,50]. After initial replication PRV enters sensory neurons and reaches the brain by retrograde spread mostly via the cranial nerves including the olfactory, trigeminal and glossopharyngeal nerve [1]. Latency is established in trigeminal and sacral ganglia and in the tonsils [16,51].

#### 4.1.3. Clinical Signs

**Figure 1:**
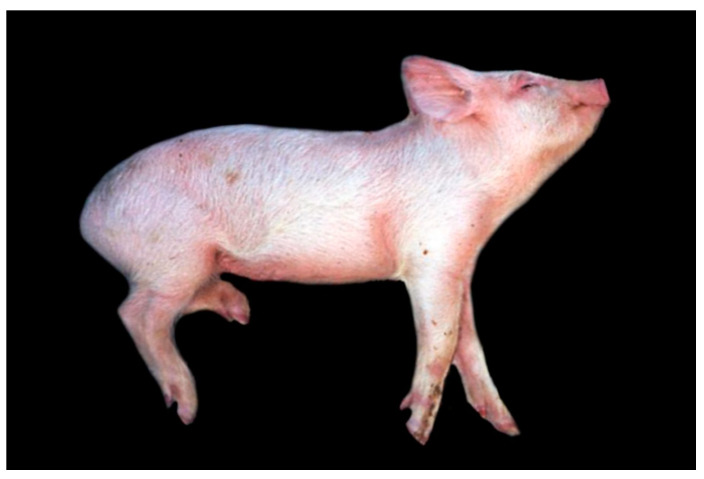
Aujeszky’s disease (AD) in piglet, showing lateral recumbency, convulsions and opisthotonus. (Courtesy: Faculdade de Veterinária, Universidade Federale Do Rio Grande Do Sul, Porto Alegre).

During the first week of life, piglets may die spontaneously without showing any specific clinical signs of AD [46]. In slightly older animals, unspecific clinical signs such as fever, anorexia, lethargy and depression are present, whereas with disease progression neurological signs predominate. These include ataxia, circling, paresis and paralysis, muscle tremor, convulsions, nystagmus or opisthotonus [15,28,38,39,46,48,52] (Figure 1). Vomiting, constipation and diarrhea are also reported frequently [15,28,39,41,43]. Pruritus can be present which can cause constant rubbing [15,39,40]. Older pigs mostly show respiratory signs including nasal discharge, sneezing and coughing [15,39]. Fetal resorption and abortion during the first month of pregnancy with macerated, mummified or dead but otherwise normal fetuses occur at later stages and are further associated with AD [29,53].

#### 4.1.4. Post-Mortem Findings

**Figure 2:**
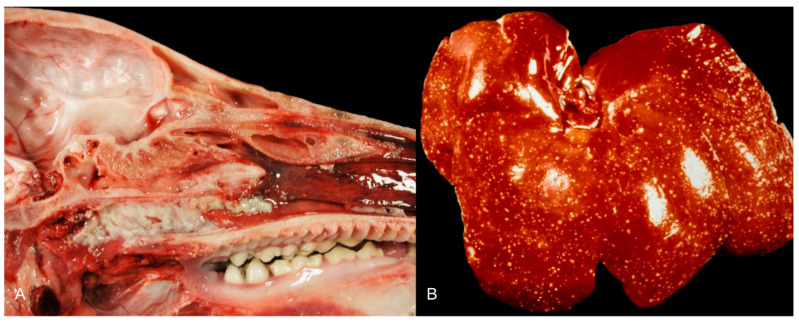
Coagulative necrosis of the palatine tonsil (**A**) and liver (**B**) of pigs infected with pseudorabies virus (PRV). ((**A**): Friedrich-Loeffler-Institut (**B**): Courtesy: Institute of Veterinary Pathology, Justus Liebig University, Giessen).

Gross changes are mostly present in young pigs [47,52]. Tiny white foci of coagulative or lytic necrosis are present in various organs including the tonsils, larynx, trachea, esophagus, liver, spleen, kidneys, adrenal glands and intestine (Figure 2). Petechiation can be detected throughout the whole body, but mainly includes the lymph nodes, lung, kidneys and brain. Congestion of the brain and lymph nodes as well as lung edema occur [28,34,38,52].

#### 4.1.5. Histopathology

**Figure 3:**
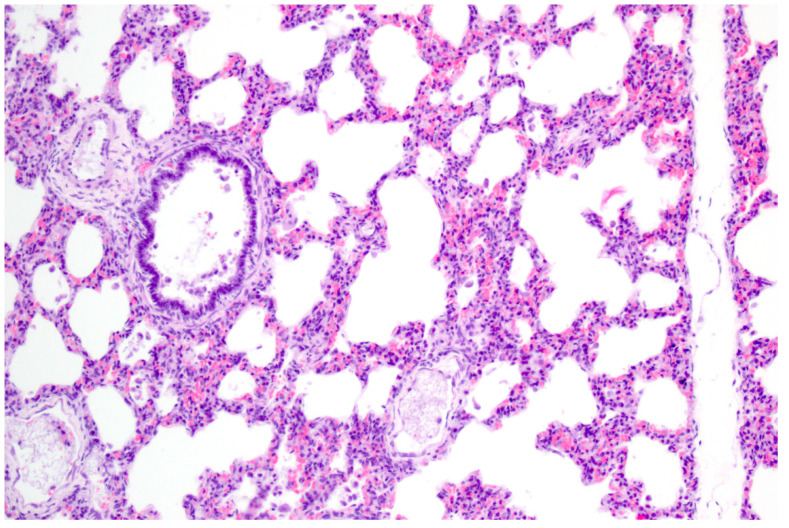
Interstitial pneumonia of a pig after natural PRV infection. The alveolar septa are slightly thickened with lymphohistiocytic infiltration. H.E. (Courtesy: Institute of Veterinary Pathology, Leipzig University, Leipzig).

PRV is pantropic which becomes obvious microscopically. Epitheliotropic lesions appear mainly in young or aborted piglets. Multifocal necrosis occurs in the liver, spleen, tonsil, lymph node, nose, lung, adrenal gland, placenta, testicle, stomach and intestine, and is often accompanied by variable numbers of amphophilic intranuclear inclusion bodies [28,38,43,44,47,52]. Hemorrhages can be present in the lymph node [28]. In some cases of naturally infected wild boar and domestic pigs either depletion or hyperplasia of lymphoid tissue is described [28,36]. Lung lesions can be either mild or severe and range from edema to interstitial pneumonia with necrosis of the epithelium, endothelium and connective tissue [34,43,47] (Figure 3). A necrotizing inflammation of arteries, veins and lymphatic vessels accompanied by thrombosis has been described after natural infection [28,38].

**Figure 4:**
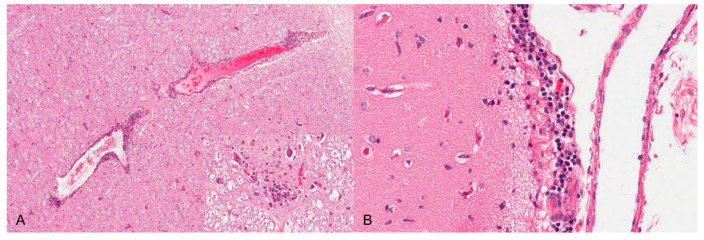
Nonsuppurative meningoencephalitis in a PRV experimentally infected piglet. (**A**) Perivascular cuffs consisting of lymphocytes and histiocytes and mild microgliosis (inset). (**B**) Lymphohistiocytic infiltration of the adjacent meninges with few neutrophilic granulocytes, H.E. (Courtesy: Department of Pathology, University of Veterinary Medicine Hannover, Hannover).

Neurotropic changes are seen in the brain, spinal cord as well as in spinal and vegetative ganglia as nonsuppurative, mainly lymphohistiocytic inflammation. The inflammatory response is characterized by neuronal necrosis and degeneration, neuronophagia and satellitosis as well as microglial activation and proliferation. Perivascular cuffs consisting of mainly lymphocytes, macrophages, and fewer neutrophils as well as meningeal infiltration are characteristic [44,46,48] (Figure 4). In piglets, panencephalitis is commonly present affecting the brainstem, cerebellum, thalamus and cerebrum including the olfactory bulb [28,44,46,48,52]. Principally, lesions are located in the grey matter, but can also involve the white matter [47,48]. Ganglioneuritis of the trigeminal, spinal, myenteric, submucous and mesenteric ganglia is common [28,43,47,52].

The detection of PRV antigen is mainly associated with degenerating or necrotic cells in multiple tissues. These include the tonsillary crypt epithelium and the reticulo-endothelial cells of lymph nodes [52]. Antigen can further be detected in the parenchyma of the liver and in the medulla of the adrenal glands. In the lung, PRV antigen is present in necrotic interstitial and peribronchial foci. Degenerating neurons and glial satellite cells are positive in the cerebral cortex [45,52]. Viral antigen is also detectable in the myenteric and submucous plexus, mesenteric, spinal and trigeminal ganglia, spinal cord, brainstem, thalamus, cerebellum and olfactory bulb [38,42,43,44,46,47,52,54]. PRV antigen can be found in the stomach and small and large intestine, but not in the mucosa [38]. Antigen is detected less frequently in the nasal or nasopharyngeal mucosa [44,52,54].

### 4.2. AD in Cattle

#### 4.2.1. General Information

Cattle are more resistant to PRV than other domestic animal species. They can be infected by direct or indirect contact with pigs [55]. If there is close contact, pigs may sniff or bite the cattle, especially in the perineum. This could also explain why naturally infected cattle usually die after intense pruritus of an area of the hind-quarters [56]. Experimental infection of cattle is possible by the intranasal (i.n.), i.m., s.c., intravenous (i.v.), and intradermal (i.d.) routes [57,58,59,60].

#### 4.2.2. Pathogenesis

In cattle, it is assumed that PRV infection takes place through injuries of skin or mucous membranes. Although the alimentary route does not generally play a role, AD after ingestion of contaminated feed is possible [61]. After experimental inoculation, PRV multiplies first in the fasciae at the inoculation site, and appears subsequently in the peripheral nerves and related spinal ganglion. It occurs within the spinal cord segment corresponding to the cutaneous entry site and finally throughout the central nervous system (CNS). The findings are indicative of a centripetal spread of virus within the axoplasm of the peripheral nerve [58]. Cattle are dead-end hosts since PRV is not transmitted directly from cattle to cattle [59].

#### 4.2.3. Clinical Signs

AD in cattle begins with undulating fever up to 42 °C until shortly before death. Characteristic for PRV are the behavioral and CNS disorders. They consist of severe, insatiable pruritus, seizure-like restlessness and later also paralysis. Incoordination, instability of gait, circular movement, howling, drooling, jaw paralysis, grunting sounds, banging heads against walls, hanging ears with excoriation at the base of the ear, nares, periorbital skin are additional clinical features [55,59,62]. Initially, there is only jerky twitching of individual muscle groups in the head, neck and back. The animals constantly beat their tails, lick or gnaw at various parts of the body, but mostly in the area of the knee folds, on the inner surfaces of the hind legs, on the udder or at the base of the tail or in the perineal area. After untying, they wander around unsteadily and then often take a dog-sitting position, rubbing (sliding) over several meters on the backside.

After experimental inoculation cattle show similar clinical signs with self-mutilation of the injection site and terminal convulsions. If there is no evidence of pruritus, spasms of the facial muscles produce retraction of the lips and salivation occurs [56].

#### 4.2.4. Sequela

As an outcome of gnawing and chafing, extensive areas of abraded skin often develop. Calves suffering from experimental AD may show signs of encephalomyelitis and die within a few hours without showing pruritus.

#### 4.2.5. Post-Mortem Findings

At necropsy there is multifocal excoriation of the skin. The affected areas of the skin are covered by a serosanguineous exudate and are markedly thickened by edema or hemorrhages extending into the subcutaneous edematous tissue and subjacent muscles. There is an increase of cerebrospinal fluid. The meningeal vessels are congested, meninges are edematous. The epi- and pericardium as well as thymus show petechiation. Most characteristically, there is pronounced pulmonary edema [55,56,57,59,62].

#### 4.2.6. Histopathology

The histological changes in the CNS consist of a nonsuppurative encephalomyelitis [55,59,60,62]. They are generally characterized by degeneration and loss of neurons with focal gliosis and neuronophagia with satellitosis, especially in the ventral horns of the affected spinal cord section and later in the pons and mesencephalon. Perivascular lymphocytic infiltrates and glial cell proliferation in the brain and spinal cord, possibly surrounded by small foci of hemorrhage can be observed. Mild infiltration can also be present in the peripheral nerves. Meningeal infiltration by neutrophils, macrophages and lymphocytes is present in the depths of the sulci. The endothelium of many of the smaller vessels of the pons and medulla can be swollen. The dorsal root ganglia of the lumbar and sacral nerves can be congested and edematous. Neuronal damage in the affected ganglia ranges from mild central chromatolysis with cytoplasmic eosinophilia to complete necrosis and ganglioneuritis with intranuclear inclusions [56,57,60,62]. Lymphocytoplasmic infiltrations of the epicardium and pericardium, thymus, kidneys and lungs are also present, eosinophilic intranuclear viral inclusions are occasionally described [57]. After experimental infection mononuclear perivascular cuffing, neuronal necrosis, focal gliosis around damaged neurons and satellitosis and neuronophagia in the cerebrum, and focal microgliosis in much of the subcortical white matter are described [57,59,60]. In addition, the neurons in the cerebrum show positive staining for PRV antigen by immunohistochemistry [55].

### 4.3. AD in Small Ruminants (Sheep and Goats)

#### 4.3.1. General Information

PRV infected sheep and goats are generally not contagious [63]. Infection usually leads to death with central nervous disorders, often with severe pruritus [63,64,65,66]. Although the frequency of natural infection in sheep is low, mortality rates of up to 60% due to AD can result in significant losses in sheep flocks [67]. Sources of infection of sheep are always infected pigs [65]. Sheep and goat are mainly infected by aerogenic spread, but are also highly susceptible to percutaneous infection [68].

Small ruminants excrete very little virus; contact infections from animal to animal are not expected. However, lambs can excrete as much virus as piglets with nasal secretion just before and when clinical symptoms appear, and horizontal transmission of PRV from lambs to pigs has been demonstrated [63].

In general, goats are more sensitive than sheep, and clinical symptoms are quite pronounced [66,69].

#### 4.3.2. Pathogenesis

After infection of the nasopharyngeal mucosa PRV multiplies at the entry point and in the regional lymph nodes and tonsils, sufficient to infect the brain and spinal cord via the olfactory bulb, glossopharyngeal nerve and other nerve tracts and causes fatal encephalomyelitis. After experimental intratracheal inoculation PRV travels from the respiratory mucosa to the central and sympathetic nervous system by two routes: (1) In the vagus and glossopharyngeal nerves to the medulla oblongata; and (2) in the postganglionic fibers to the sympathetic ganglia. The presence of virus in the nasal mucus indicates that horizontal transmission of pseudorabies virus may occur among sheep [64]. In addition, following lympho-hematogenic spread, the virus is transported to the CNS, spleen, liver, lungs, kidneys, salivary glands as well as to mucous membranes of the respiratory, digestive and genital tracts.

#### 4.3.3. Clinical Signs

Sheep of all ages are affected, which develop the clinical picture of central nervous irritation and paralysis [63,64,65,69]. The main clinical signs are fever, restlessness and movement disabilities. A pathognomonic sign is the increasingly pronounced, insatiable itching. Paresis, lateral recumbency, paralysis of the pharynx, dyspnea, rumen atony characterize this form of central nervous paralysis [70,71].

Lambs collapse and die peracutely [71]. In adult animals, CNS signs, ptyalism, loss of voice, difficulty swallowing and eventually pharyngeal paralysis are more pronounced. The body temperature is not always elevated, fever might occur only at the beginning of the disease and is not severe [64,67,70,71].

#### 4.3.4. Post-Mortem Findings

Gross pathology is generally inconspicuous involving skin injuries after facial trauma, wool-free areas of the lateral chest behind the elbow joint, abdominal wall and on the limbs, wool in the rumen, pulmonary edema with lobular interstitial pneumonia, hyperemia of the meninges, petechiae in the cervicothoracic ganglia and dilated esophagus are described [63,64,65]. Ganglioneuritis with intranuclear inclusions is rather typical, but not always present. Nonsuppurative encephalomyelitis with inflammatory-proliferative and degenerative neurons, partly with intranuclear inclusion bodies, neuronophagia and focal gliosis are only recorded in half of the cases [28,66]. Sometimes severe acute multifocal necrotizing bronchopneumonia with necrotizing vasculitis and intranuclear inclusion bodies within the neurons of the cervicothoracic ganglia occurs [72]. Experimental studies and field cases suggest that the medulla oblongata and the trigeminal ganglia are important tissues to harvest for histologic and virus isolation procedures. Additional data further indicate that the cranial cervical and cervicothoracic ganglia are beneficial as tissues for the diagnosis of pseudorabies in sheep [73].

### 4.4. AD in Dogs and Cats

#### 4.4.1. General Information

AD in carnivores is usually associated with the consumption of raw pork from infected animals, or by contact between dogs and infected swine or carcasses. Especially for hunting dogs, PRV is a serious risk [14,74,75,76]. The pathogenesis of AD in carnivores is not well understood.

#### 4.4.2. Clinical Signs

**Figure 5:**
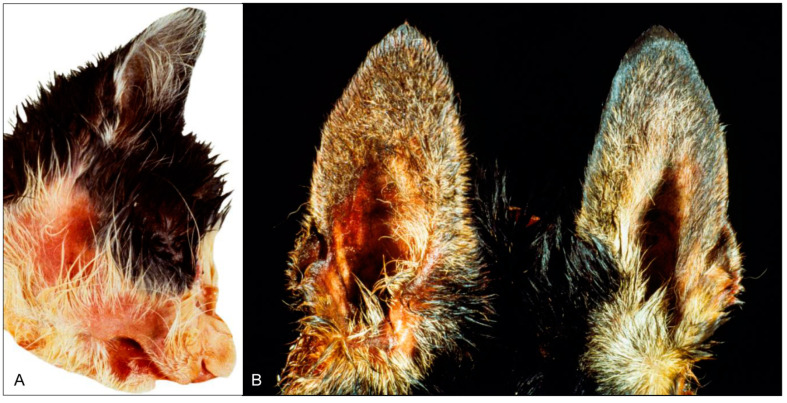
Facial skin erosions in a cat (**A**) and a dog (**B**), both naturally infected with PRV. (Courtesy: Institute of Veterinary Pathology, Justus Liebig University, Giessen).

AD in dogs and cats is frequently acute and leads to death shortly after the onset of clinical signs. At the beginning of disease, rather unspecific clinical signs are predominant including anorexia, fever, depression and lethargy [77,78,79,80,81,82,83,84,85]. Vomiting is frequently reported in dogs and cats [75,76,78,79,80,81,82,83,84,85,86], whereas diarrhea is only described in dogs [75,76,80]. Excessive salivation is also present in both species [78,79,80,81,83,84,85]. One of the most prominent clinical features is intense pruritus, which develops mainly at the head and is followed by self-mutilation, severe skin abrasions and edematous reaction in the affected area [75,78,79,80,82,83,84,85,86,87] (Figure 5). However, itching can be absent in animals suffering from AD [76,77,79,81,88]. Neurological impairment such as ataxia, tremor or head pressing is also reported [76,77,78,83,84,85,87,88]. Spasms of the musculature including trismus as well as vocalization, aggressiveness and dyspnea can be present in dogs and cats [75,76,77,78,79,80,83].

#### 4.4.3. Post-Mortem Findings

In naturally infected animals, second to skin lacerations and edematous, hemorrhagic subcutaneous tissue, hemorrhages are observed in different organs, primarily in the heart and in the lung [75,76,78]. Hemorrhages occur in experimentally infected dogs irrespective of the inoculation routes [78,89]. In detail, the animals show petechiae and ecchymoses in the epi-, myo- and endocardium as well as valvular hemorrhages. However, the pathogenesis is not clear. It has been suggested that cardiac damage results from increased sympathetic stimulation to myocardial cells and/or endothelial cell disruption due to inflammation of the stellate ganglion [89]. Also secondary cardiovascular complications often result from brain injury and result into a systemic catecholamine storm, which eventually leads to vasoconstriction and an increased myocardial workload and oxygen demand [90]. Necrosis of myofibers and hemorrhages then follow. In contrast, hemorrhages in the thymus are either of neurovascular or agonal origin [78,91]. In dogs, hemorrhages with “black content” can be found in the stomach (acid hematin) and small intestine (melaena) [75,78,80,82,85]. In experimentally infected dogs inflammation of the small bowel has been reported [78]. In line with this, mesenteric lymph nodes are enlarged [75]. After natural infection, hemorrhages further appear in the pleura and kidney [78]. Frequently, pulmonary congestion and edema, as well as pericardial, thoracic and peritoneal effusion, and meningeal congestion occur in naturally and experimentally infected animals [76,78,80,85,89]. Animals lacking gross lesions are described as well [81,92]. 

#### 4.4.4. Histopathology

**Figure 6:**
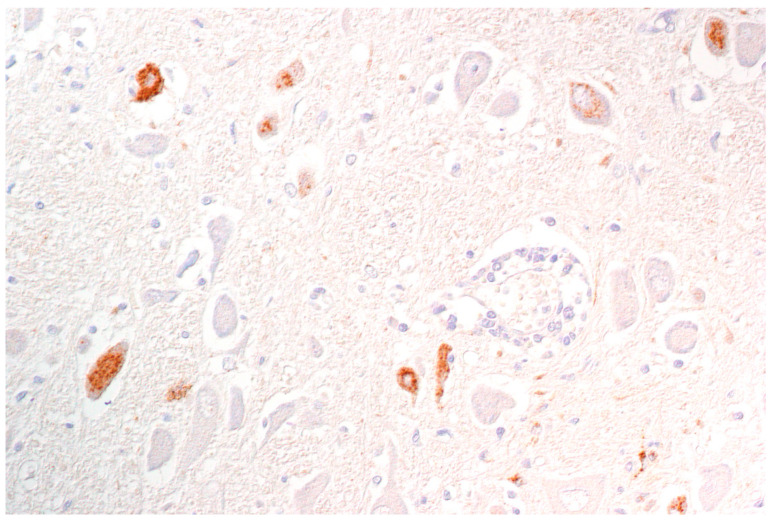
Dog, brainstem, PRV antigen detection in neurons and few glial cells. (Courtesy: Institute of Veterinary Pathology, Leipzig University, Leipzig).

**Figure 7:**
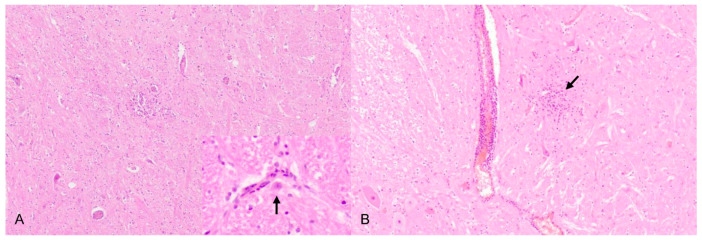
Nonsuppurative brainstem encephalitis. (**A**) Dog, mild lymphohistiocytic perivascular and parenchymal infiltrates, neuron with intranuclear inclusion bodies, Cowdry type A (inset, arrow). (**B**) Cat, moderate perivascular infiltration and microgliosis (arrow), H.E. (Courtesy: Clinical and Comparative Neuropathology, Centre for Clinical Veterinary Medicine, Ludwig-Maximilians-Universität, Munich).

**Figure 8:**
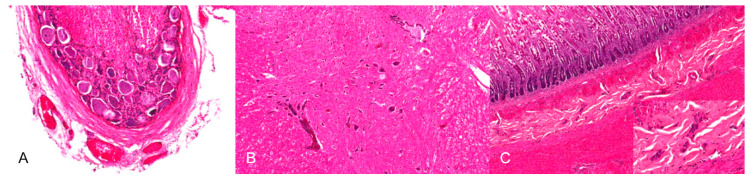
Inflammation of neural tissues of a PRV-infected dog. (**A**) Moderate, lymphohistiocytic ganglionitis with neuronal damage, neuronophagia and gliosis. (**B**) Mild, multifocal lymphohistiocytic myelitis. (**C**) Inflammation of the myenteric plexus. The ganglia of the myenteric plexus are infiltrated by lymphocytes and histiocytes (inset). (Courtesy: Institute for Veterinary Disease Control, Mödling).

**Figure 9:**
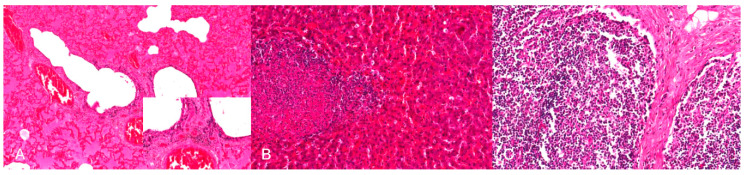
Inflammation and necrosis in different organs in a PRV-infected dog. (**A**) Mild lymphocytic bronchiolitis with severe, diffuse alveolar edema. (**B**) Focal hemorrhagic necrosis in the liver. (**C**) Multifocal necrosis of lymphocytes in the lymph node resulting in lymphoid depletion, H.E. (Courtesy: Institute for Veterinary Disease Control, Mödling).

By immunohistochemistry, PRV antigen is mainly present in neurons and glial cells of the brainstem, trigeminal ganglia, spinal cord and abdominal vegetative plexus such as the myenteric plexus [75,76,85,87] (Figure 6). Further, cardiomyocytes as well as fibrocytes and glandular epithelium of the stomach carry PRV antigens [76]. This corresponds to observations in a cat showing antigen positive cells in the intestinal tract and in sympathetic lumbar ganglia [84]. In experimentally inoculated dogs antigen is also present in the pancreas and parotid gland [93], and in the sympathetic stellate, celiac and caudal mesenteric ganglia [78]. Mild to severe nonsuppurative encephalitis mainly affects the brainstem [28,75,76,78,81,84,85,86,87] (Figure 7). Inflammation of the cerebrum [85,88], midbrain and cerebellum [28] may also occur. The inflammatory response is characterized by neuronal necrosis of brain parenchyma and perivascular cuffs consisting of mainly lymphocytes and macrophages. There is glial cell proliferation in the grey and white matter, satellitosis, neuronophagia and more or less intralesional eosinophilic intranuclear inclusion bodies. The adjacent meninges are infiltrated by mononuclear cells, and endothelial cells can be hypertrophic [86]. Inflammatory infiltrates are further detectable in trigeminal ganglia, spinal cord, abdominal ganglia and vegetative plexus as well as in the adrenal gland [75,80,82,84,85] (Figure 8). The heart, lung, kidney, liver, intestinal tract, tonsil are also affected by inflammation, necrosis or hemorrhage [78,81,84,86] (Figure 9). Degeneration and necrosis of cardiomyocytes with mild or no inflammatory reaction can occur in dogs. Focal necrotic areas are evident in the liver, whereas lymphoid depletion as well as hemorrhages are seen in the thymus and lymph nodes (Figure 9). Especially in cats, tonsillary necrosis of the crypt epithelium is reported [84,92]. In scratched skin regions necro-ulcerative dermatitis with subcutaneous edema are present [75,78].

### 4.5. AD in Wild Carnivores

Several cases of natural AD have been reported in wild carnivores including fourteen wild foxes [22,94,95], over 1200 captive foxes [96], about 8000 captive mink [97], one Iberian lynx [98], three wolves [99], three captive coyotes [100], four brown bears [101], one black bear [102], one Florida panther [23] and six raccoons [103]. Experimental studies were carried out in blue foxes [104] and three raccoons [103].

Clinically, wild foxes present with neurological signs such as rolling or excessive biting of branches, paralysis, headshaking and ataxia. Pruritus and skin abrasions are present in the majority of foxes and appear predominantly at the head and limbs [22,95]. Additionally, fever, anorexia, salivation, vomiting, diarrhea, dyspnea, vocalization and apathy are reported [22,96]. Macroscopically, except for alopecic skin [22,95] and in one case hemorrhagic gastritis and enteritis [94], gross lesions are absent. In twelve foxes PRV could be isolated from different brain regions [22]. PRV is mainly detectable in the brainstem and cerebellum and to a lesser extent in the cerebrum and cervical spinal cord. Experimentally inoculated blue foxes show unspecific clinical signs such as anorexia, depression and coma. Nonsuppurative encephalitis accompanied by neuronal necrosis, neuronophagia and perivascular cuffs is seen in the brainstem and cervical spinal cord, which are also positive in immunohistochemistry [104].

For an Iberian lynx, alopecia in the neck region, black content in the stomach and intestine and meningeal congestion are reported [98]. Histopathologically, diffuse inflammatory and necrotic changes were present in the brain. Meningoencephalitis characterized by perivascular accumulations of mononuclear cells of mainly lymphocytes were observed as well as multiple foci of microgliosis, satellitosis, neuronal degeneration and neuronophagia. The cerebrum and cerebellum showed foci of demyelination. Consistent with gross lesions, histopathologically necrotizing inflammation affected the intestinal tract.

Three wolves presented with neurological signs including ataxia, abnormal head posture and circling. No gross lesions appeared except for pulmonary congestion. PRV diagnosis was confirmed by real-time PCR. Histopathology was not available in this case [99].

Three captive coyotes showed vocalization and anorexia. At necropsy, alopecic, edematous and hemorrhagic areas were found at the head as well as black intestinal content. Histopathological examination revealed multifocal nonsuppurative meningoencephalitis characterized by hemorrhage, necrosis, microgliosis, perivascular cuffs, and intranuclear inclusion bodies [100].

A Florida panther showed peritoneal and thoracic effusions, dark red content in the intestinal tract, intestinal erosions as well as meningeal congestion [23]. PRV was confirmed by virus neutralization test and immunofluorescence assay.

AD was detected in four captive brown bears which showed anorexia, depression, salivation, dyspnea, severe pruritus, self-mutilation, dyspnea and paralysis [101]. Macroscopically, only skin abrasions were detectable. AD was confirmed by serum neutralization test and restriction fragment length polymorphism. In line with this, AD has also been reported in a black bear [102]. The bear was lethargic, depressed and anorectic. Neurological deficits such as ataxia and shaking as well as vocalization occurred prior to death. Macroscopically, pulmonary congestion and edema were present as well as aspirated ingesta. The animal had ascites and volvulus of the small intestine with congestion of mesenteric lymph nodes. Histopathologically, lungs showed edema and smooth muscle hyperplasia. The liver was congested and necrotic and contained intranuclear eosinophilic inclusion bodies. Hemorrhages, necrosis and inclusions were also detected in the adrenal gland. Viral antigen was not detectable in the brain.

AD in raccoons has been confirmed after natural and experimental infection [103]. The clinical signs of raccoons experimentally infected with PRV include anorexia, salivation, vomiting, pruritus and neurological signs such as convulsions and pharyngeal paralysis. At necropsy, there were congestion of lungs, cardiac hemorrhages and meningeal congestion. Viral antigen was found in the tonsils, brainstem, cerebellum and cerebrum.

Recently, over 8000 farmed mink died in China within one week [97]. The animals showed typical clinical signs for AD including pruritus and self-biting as well as vomiting, diarrhea and dyspnea. At necropsy, multifocal hemorrhages were present on heart, kidney and lung. The intestine was reddened and there was congestion of the spleen and pulmonary edema. PRV was isolated from brain tissue.

### 4.6. AD in Horses

The horse, likewise the donkey and the mule, are extremely rarely infected by PRV [105]. In the Netherlands, PRV was isolated from the brain in two independent natural cases of AD with severe neurological signs, including excessive sweating, muscle tremors, licking and chewing at objects, and periods of mania. There was a nonsuppurative meningoencephalitis by histopathological investigation [106]. By immunohistochemistry, DNA-in situ hybridization and serological tests PRV antigen and DNA were detected in neurons of the cerebrum. For one of these cases the isolated virus was inoculated into the conjunctiva and nostrils of two ponies [107]. These horses developed fever 7 days after inoculation and started to show abnormal behavior, with severe neurological signs on day 9 post infection (p.i.). One pony became excited and the other one was depressed. One pony died on the ninth day after inoculation and the other was humanely put to death on day 10 p.i. Both ponies developed PRV neutralizing serum antibodies. The virus was recovered from several parts of the brains and the eyes. It was therefore concluded that AD in horses fulfils Koch’s postulates [107]. However, in principle, horses are regarded as highly resistant to PRV infection [1].

### 4.7. AD in Chickens

PRV was experimentally tested in chickens [108,109]. Poultry are susceptible to infection, which is age-, dose- and virus strain-dependent. Chickens can be infected via different routes, including intracerebral (i.c.), i.m., s.c., i.n., intraocular (i.o.) and via skin scarification. Intracerebral inoculation leads to 100% mortality in one-day-old chickens and declines in older birds, dependent on the inoculation dose. Chickens older than two days do not develop disease after intramuscular inoculation. Animals which survive an intracerebral or intramuscular infection are resistant to reinfection. There exists only one case report of natural infection in thousands of three- and four-day-old chickens which died of AD [110]. The birds showed recumbency, excitation and paralysis. PRV was isolated from the brain of dead birds from inoculated eggs and then tested in chickens up to seven days of age. Intracerebral, intraperitoneal and intramuscular inoculation led to death, but oral, eye drop or spray application did not result in disease. Affected birds revealed meningitis, edema, perivascular cuffing and hemorrhages mainly in the cerebrum and spinal cord. Also inclusion bodies were detected in ganglion cells in the spinal cord. This outbreak of AD in chickens was due to a contaminated vaccine against Marek’s disease.

### 4.8. AD in Laboratory Animals

Although AD does not occur in laboratory animals under natural conditions, they are highly susceptible to experimental infection [111]. Rabbits were found to be the most sensitive species [112] and have been used for diagnostic purposes [113]. When subcutaneously inoculated, rabbits exhibit neurological deficits, hyperactivity, intense pruritus and self-mutilation at the site of inoculation [114]. Macroscopically, rough fur and skin abrasions with serofibrinous exudation can be observed [28,114]. However, only few histopathological data exist about PRV infection in rabbits. One study shows inflammatory mononuclear reaction in the skin lesions accompanied with extensive necrosis affecting the epidermis, dermis and muscles. Moreover, suppurative inflammation as well as coagulative necrosis are observed in the liver, and there is pulmonary congestion and edema [28]. The brain, spinal cord as well as spinal ganglia are unaffected.

**Figure 10:**
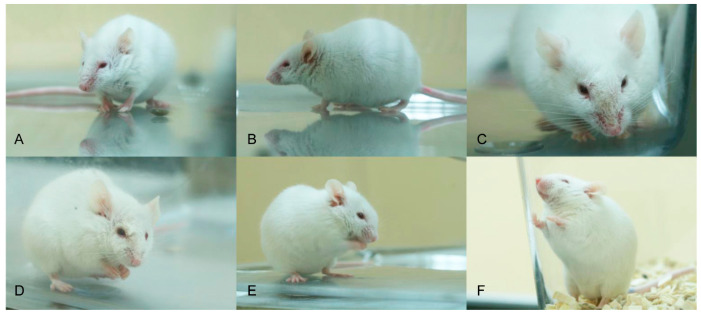
Mice intranasally infected with PRV showing hunching (**A**), ruffled fur (**B**), facial dermal erosions (**C**), unilateral conjunctivitis (**D**), nasal bridge edema (**E**) and dyspnea (**F**).

**Figure 11:**
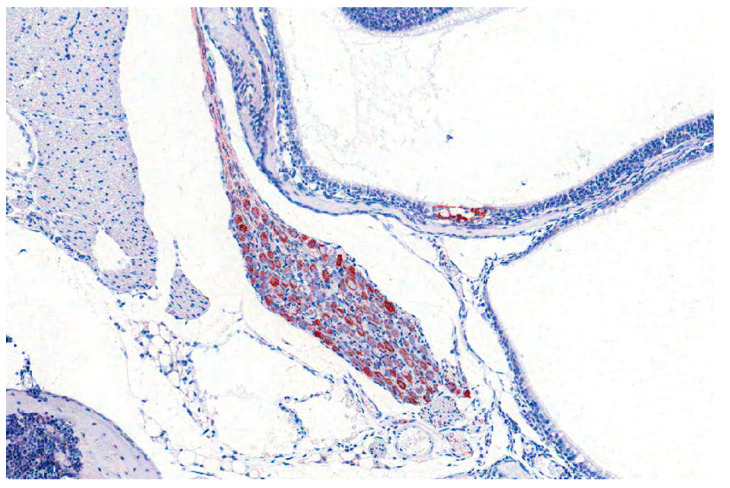
Pterygopalatine ganglion of a mouse infected experimentally with PRV. PRV-antigen reveals numerous positive neurons (red labelling by immunohistochemistry).

Rats and mice are less susceptible than rabbits. Compared to mice, rats display a greater resistance to PRV infection, since successful oral inoculation requires 10^6^ PFU and mucosal injuries. In mice, 10^4^ PFU is the minimal infectious dose, dependent on the virus strain [111]. Mice can be infected by various inoculation routes including: i.n., p.o., i.o., i.c., i.v., i.m., intraperitoneal (i.p.), s.c. or via foot pad [24,25,27,115,116,117,118,119,120,121]. Depending on the inoculation route, the incubation period of AD in mice is longest after intranasal or intraocular infection [115]. After intracerebral infection, mice are recumbent and comatose and die within two days post infection (p.i.). A generalized pruritus can be observed after intravenous and intraperitoneal inoculation with phases of frequent „face-washing“ [115]. With disease progression pruritus and edema at the head worsen, which is followed by collapse of the animals. Mice infected intranasally do not show skin lesions, but appear to be blind. They also show violent, spasmodic movements before they die. This is in contrast to previous experiments in mice, where intranasal infection leads to a more severe clinical picture [24,25,27,121]. Mice show hunching, anorexia, depression, hyperactivity and severe pruritus at the head (Figure 10). Some develop excitations, convulsions and dyspnea. The animals have hemorrhagic dermal erosions and ulcerations, nasal bridge edema and acute catarrhal conjunctivitis and do not survive longer than 3 d p.i. The same is true for subcutaneous (flank) inoculation in mice [118]. After intramuscular injection, mice exhibit intense pruritus at the inoculation site, collapse and die. Excessive itching, head edema and self-mutilation are also present in mice after intraocular infection [115]. Virus is only detectable in the brain after i.c. or i.o. infection. The spleen shows viral antigen positive cells after i.v. infection, whereas PRV can be found in the kidney after i.m., i.v. and i.p. and to some extent after i.n. infection [115]. In contrast, Klopfleisch et al. [25] did not identify viral antigen in other tissues than the brain and epithelial cells in the nasal mucosa after i.n. infection. Immunohistochemically, PRV antigen is localized in the respiratory and nasal gland epithelium, trigeminal ganglion, brainstem and to some extent in the midbrain. Further, the sympathetic superior cervical ganglion as well as the parasympathetic pterygopalatine ganglion are affected [24,25] (Figure 11). In addition, the olfactory epithelium, the vomeronasal organ as well as blood vessel walls are positive for viral antigen [121] as well as salivary glands [27]. Inflammation is only mild and occasionally present in the nose and superior cervical ganglion [27]. Subcutaneous inoculation results in infection of the brainstem and the cerebellum [118], whereas after foot pad inoculation virus can moreover be isolated from the sciatic nerve, lower spinal cord, adrenal gland, coeliac ganglia and paravertebral ganglia [119]. This is in line with inflammatory changes in dorsal root ganglia and at the site of inoculation [120].

Like mice, rats are infectable by different inoculation routes [115,116,122] and show similar clinical signs. Diarrhea as well as orchitis are reported for male rats [115]. After intramuscular injection PRV can be isolated from the inoculation site, kidney and brain. Nonsuppurative encephalitis, perivascular cuffing in the grey and white matter, slight neuronal necrosis of mitral and tufted cell layers in the olfactory bulb and few intranuclear inclusion bodies are present in rats infected orally, subcutaneously and intranasally. Endothelial hypertrophy also occurs, but these lesions are not attributable to a certain inoculation route [116]. After intraperitoneal injection, PRV antigen is detectable in the peritoneum, myenteric and submucous plexus, abdominal sympathetic and mesenteric ganglia, dorsal root ganglia, spinal cord and adrenal gland [117]. Intraocularly infected rats have been mainly used in PRV research as a well-characterized model of PRV infection [122,123,124]. In this model, viral antigen can be reproducibly detected in retinal ganglia as well as in the cerebrum, midbrain and superior cervical ganglia.

PRV in guinea pigs has not been studied intensively. Guinea pigs can be infected via the intramuscular and intranasal routes [125]. The animals show fever and display severe pruritus, self-mutilation and neurologic deficits such as paresis and epileptiform convulsions. Histopathologically, necrosis of neurons in the olfactory bulb and occasionally in the cerebral cortex, hippocampus, midbrain and brainstem are observable. Intranuclear inclusion bodies are only occasionally seen. Few foci of microgliosis as well as perivascular cuffing mainly consisting of lymphocytes and macrophages appear in affected brain areas.

Rhesus macaques exhibit a greater resistance to PRV infection [126]. Intradermal (i.d.), i.m. and i.v. inoculation do not cause disease unlike i.c. or intrasciatic infection. In the latter, most of the animals show fever and occasionally develop neurological signs within one week after inoculation. When neurological signs are definite, the monkeys do not recover from the disease. In macaques, PRV is strictly neurotropic, since the virus is not present in other tissues than the nervous system. After i.c. inoculation, virus is found in both cerebral hemispheres as well as in the lumbar spinal cord. In animals inoculated via the intrasciatic route PRV infects the lumbar and cervical spinal cord, the brainstem and the frontal, temporal and occipital lobe, depending on how long the animals survive. Following intracerebral inoculation neuronal degeneration is mainly observed in the cerebral cortex, whereas basal ganglia and the brainstem are infiltrated by immune cells. The cerebellum is unaffected. In non-fatal cases infection leads to porencephalitic cavities of the brain. After intrasciatic inoculation, neuronal necrosis occurs in the spinal ganglia accompanied by inflammation. Similar changes are present in the spinal cord as well as in the brain compared to intracerebral inoculation.

### 4.9. PRV Infection in Humans

PRV infection in humans has been discussed controversially. In 1970, Jentzsch and Apostoloff [17] reported that even after self-inoculation, AD does not occur in humans. While the virus caused no clinical signs after s.c. injection in two independent experiments, inoculation in the rabbit was fatal. However, first cases of PRV infection were reported in 1914 in two technicians exposed to a laboratory cat infected with PRV [127]. Both patients had injuries on their hands and experienced pruritus and swelling of the wound.

Some years later, two lab workers with injuries from necropsy and handling of a PRV-infected dog, respectively, showed pruritus and erythema of the site of injury and aphthous stomatitis [128]. A rabbit was inoculated with serum from a single patient and developed AD.

Hussel et al. [129] reported two animal handlers, one night watchman and one veterinarian with weakness and throat pain. All four patients worked on a pig farm and were exposed to a PRV-infected dog.

PRV was confirmed in three patients who had close contact to cats [130]. One patient cut his thumb when he washed the dishes of a PRV-infected cat. Clinical signs were comparable in all patients, but more severe in the patient who had the injury. Unspecific clinical signs such as weakness, fever, sweating, tiredness or diarrhea characterized the beginning of the disease. Dysphagia was present in all three patients, but the person with the thumb injury moreover showed dysgeusia, glossodynia and pain of muscles and joints, hypersalivation, headache, tinnitus and weight loss. In all three patients anti-PRV antibodies were detected.

In 1992, an outbreak of AD in cattle was reported [131]. Although cattle are considered dead-end hosts, six people who worked on the farm experienced pruritus of palms, lower and upper arms, shoulder and back, but were not tested for PRV infection.

In contrast, one patient exposed to sewage on a Chinese hog farm developed fever, headache and visual impairment and was diagnosed for endophthalmitis caused by PRV, which was detected in the vitreous humor via next generation sequencing (NGS) [132]. Anti-PRV antibodies were found in the serum.

Likewise, retinitis or retinal necrosis was diagnosed in Chinese patients who had contact to raw pork [133,134]. In two others, lesions were visible in the limbic system, basal ganglia and in the midbrain with magnetic resonance imaging (MRI). The patients presented with fever, convulsions, loss of consciousness and respiratory failure.

Comparable findings were observed in a pig slaughterer, a pig handler and three pork cutters, three of whom had hand injuries [135]. MRI revealed lesions in the frontal lobe, insular cortex, temporal lobe and further in the caudate nucleus, cingulate gyrus, thalamus and basal ganglia. In two out of five patients the optic nerve atrophied and in one patient PRV was detected in vitreous humor. PRV was detected in all other patients in cerebrospinal fluid (CSF). Clinically, the affected persons showed headache, fever, visual impairment, convulsions, respiratory failure, mental status changes, memory loss or incontinence.

A similar case of PRV infection was reported in a veterinarian who suffered a hand injury during pig necropsy [136]. The patient had fever, headache and seizures and showed abnormalities in the basal ganglia, occipital lobe, limbic lobe and thalamus.

## 5. Summary and Conclusions

PRV as a primarily porcine pathogen can infect a wide range of mammals. Although self-inoculation did not result in disease in men [17], several cases have been reported in China recently [18]. Affected people usually had skin injuries and dealt with pigs or pork. Lesions were localized to the cerebral cortex including the limbic system and insular cortex within the temporal and frontal lobe, respectively, which is comparable to herpes simplex encephalitis [137]. However, no live virus has been isolated so far from infected patients, but has only been detected via NGS or indirectly by serology. Therefore, and since only few infections have occurred so far, PRV is still not considered a ‘true’ zoonotic pathogen.

In all other animal species, although the clinical picture of AD is largely comparable, there are some notable differences regarding the clinical signs, affected organ systems and distribution of inflammatory response.

In pigs, the pathogenesis of AD and pathways of neuroinvasion are better understood as compared to other mammals. While in piglets PRV has a multi-organ distribution, the disease is only limited to the respiratory and reproductive tract or is even asymptomatic in older animals. In addition to fever and other non-specific clinical signs, piglets show severe neurological signs or suffer from diarrhea or vomiting. Pigs develop pruritus less frequently compared to non-porcine species. In piglets, PRV causes a panencephalitis. Consequently, the presence of PRV antigen is not attributed to a specific location in the brain unlike in other mammals. Since PRV results in systemic infection, also including the intestinal tract, different vegetative abdominal plexus and ganglia are affected.

In dogs and cats, the infection is comparable to pigs as several organs are affected. Especially in dogs, hemorrhages of the heart, thymus and gastrointestinal tract occur coinciding with inflammation. PRV infection in carnivores is assumed to result from ingestion of infected or contaminated raw pork and in contrast to pigs is not associated with viremia. After ingestion, the virus might replicate in the oral and gastrointestinal mucosa, and is then transported within axons to the central nervous system. By this, abdominal plexus and ganglia as well as trigeminal ganglia are commonly infected. Dogs and cats often show diarrhea and vomiting, which might reflect that PRV preferably infects sympathetic cells of intestinal ganglia with subsequent inflammation. The infection of predominantly peripheral sympathetic neurons by PRV also applies to other animal species. The severity of neurological signs varies and pruritus leading to self-mutilation is very common in dogs and cats. Unlike pigs, meningoencephalitis in dogs and cats is rather confined to the brainstem and spinal cord, which might be due to the short time until death after infection or might be explained by the route of infection. PRV infection in wild carnivores is largely comparable to dogs and cats.

In cattle, PRV infection is mainly restricted to the nervous system, and viral antigen or other lesions are rarely found in other tissues. In the field, it is thought that PRV results in the infection of sacral and lumbar nerves and dorsal root ganglia, but also of the brain. It seems that manifestation of infection depends on the entry site of PRV. Experimental data show an inoculation route-dependent distribution of the infection. The more cranially the cattle are inoculated, the more widespread the infection of the brain. In small ruminants, PRV infection leads to a disease similar to that in cattle, but infection more frequently involves the lungs.

Laboratory animals, except for rhesus monkeys, are highly susceptible to PRV infection. Particularly the rabbit succumbs to death even after receiving low doses of PRV. Several cases of natural infection with the alphaherpesvirus herpes simplex virus 1 (HSV-1) have also been described in rabbits [138,139], supporting that this animal species is highly susceptible to alphaherpesviral infection. Why and how rabbits are more vulnerable to herpesviral infection remains unresolved yet.

In contrast, rhesus monkeys are less sensitive to PRV infection, but when inoculated in nervous tissue, they can show severe lesions primarily located in the frontal and temporal lobe as compared to humans suffering from HSV-1 encephalitis or suspected PRV infection [18,137]. This indicates a neural tissue-specific tropism of alphaherpesviruses towards the frontal and temporal lobes.

In rats and mice, depending on the inoculation route, PRV infects the neuronal region, which receives fibers from or project to the site of inoculation. Thus, rats and mice are used as standardized animal models either to study alphaherpesviral neuroinvasion, the host’s response towards infection [24,25,140], or to analyze and identify neuroanatomical networks [141]. PRV mouse models can also be helpful to provide insights into the mechanisms underlying human alphaherpesvirus induced diseases such as HSV-1 encephalitis [27] or neuropathies after infection with the closely related varicella zoster virus [142].

## Supplementary Materials

The following are available online at https://www.mdpi.com/2076-0817/9/8/633/s1, Table S1 Literature overview of Pseudorabies in different species.
