# Supplementary material for: Comparative Pathology of Pseudorabies in Different Naturally and Experimentally Infected Species—A Review
Source: Pathogens. 2020 Aug 4;9(8):633. doi: 10.3390/pathogens9080633 (PMC7460128; doi:10.3390/pathogens9080633)
Supplement: Supplementary file 1 [file pathogens-09-00633-s001.pdf]

Table 1. Literature overview of Pseudorabies in different species.

<u>Abbreviations</u>

C: Congestion	p.o.: Per oral
CSF: Cerebrospinal fluid	I: Inflammation
d: Days	i.c.: Intracranial
D: Degeneration	i.d.: Intradermal
E: Edema	i.m.: Intramuscular
G: Gliosis	i.n.: Intranasal
H: Hemorrhage	i.o.: Intraocular
LD: Lymphoid depletion	i.p.: Intraperitoneal
LH: Lymphoid hyperplasia	i.t.: Intratracheal
m: Months	i.v.: Intravenous
MRI: Magnetic resonance imaging	IN: Intranuclear inclusion bodies
N: Necrosis	s.c.: Subcutaneous
N/A: Not available	VA: Viral antigen detection
NGS: Next generation sequencing	w: Weeks
o.n.: Oro-nasal	y: Years

Pigs, natural infection

Reference	Clinical signs	Gross pathology	Pathohistology
Verin *et al*. 2014<br><br>Wild boar	None	None	Tonsil: VA<br>Lymph node: VA
Lari *et al.* 2006<br><br>Wild boar	N/A	N/A	Tonsil: N, VA<br>Spleen: LH and LD<br>Lymph node: LH and LD, VA<br>Lung: I
Capua *et al.* 1997<br><br>Wild boar	N/A	Throat/chest edema	N/A

Reference	Clinical signs	Gross pathology	Pathohistology
Ezura *et al.* 1995<br><br>4 domestic pigs	Incoordination<br>Tremor	Meningeal congestion	CNS (N/A): N, VA<br>Tonsils: N, IN<br>Lung: VA<br>Liver: N, IN, VA<br>Adrenal gland: N, IN, VA<br>Stomach: N, VA<br>Small intestine: N, VA<br>Large intestine: N, VA<br>Myenteric plexus: N, IN, VA<br>Submucous plexus: N, IN, VA<br>Skin: I, D, IN, N, VA<br>Subcutaneous vessels: I
Olander *et al*. 1966<br><br>30 domestic Pigs	Fever<br>Anorexia<br>Vomiting<br>Constipation<br>Staggering<br>Convulsions<br>Restlessness<br>Depression	Lymph node: H, C<br>CNS:C<br>Lung: consolidation	Cerebrum: I (+++), N<br>Brainstem: I (+++), N<br>Cerebellum: I (+) N<br>Spinal cord: I (+), N<br>Trigeminal ganglion: I, N<br>Lymph node: H, LH, I<br>Vessels: I<br>Lungs: C, E<br>Spleen: LD

Pigs, experimental infection

Reference	age	Inoculation route	Clinical signs	Macroscopy	Pathohistology
Verpoest *et al*. 2017<br><br>23 domestic pigs	1) 2 w<br>2) 15 w	i.n.	1) Fever<br>Depression<br>Diarrhea<br>Vomiting<br>Nasal discharge<br>Dyspnea<br>Circling<br>Paresis<br>Trembling<br>2) Anorexia<br>Depression<br>Fever<br>Dyspnea<br>Neurological deficits	N/A	N/A
Müller *et al.* 2001<br><br>4 wild boar	9-11 w	i.n.	Fever<br>Anorexia<br>Depression<br>Dyspnoea<br>Sneezing<br>Coughing<br>Salivation<br>Conjunctivitis<br>Pruritus<br>Vomiting<br>Blindness<br>Circling<br>Convulsions<br>Paresis	N/A	N/A
Kimman *et al.* 1994<br><br>8 domestic pigs	10 w	i.n.	Ataxia<br>Paralysis<br>Pruritus	N/A	N/A

Reference	age	Inoculation route	Clinical signs	Macroscopy	Pathohistology
Kritas *et al.* 1994<br><br>2 domestic pigs	4-5 d	i.n.	Anorexia<br>Depression<br>Ataxia<br>Vomiting	N/A	Nasal epithelium/nasal glands/nerve fibers: VA<br>Trigeminal ganglion:VA<br>Brainstem: VA<br>Cerebellum: VA<br>Thalamus: VA
Narita *et al.* 1991<br><br>8 domestic pigs	1-4 w	i.n.	N/A	Nose: filled with pus	Nose: I, N, VA<br>Olfactory nerve: I, D, VA<br>Olfactory bulb: I, VA
Wittmann *et al.* 1983<br><br>4 domestic pigs	8-12 w	i.n.	Fever<br>Anorexia<br>Nasal discharge<br>Loss of voice<br>Depression	N/A	N/A
Narita *et al.* 1990<br><br>8 domestic pigs	1-4 w	i.n.	Anorexia<br>Ataxia<br>Tremor<br>Nystagmus	Hemorrhages throughout the body<br>Liver: white foci<br>Brain: C<br>Kidney: H	Liver: N, IN, VA<br>Spleen: N, IN<br>Lung: N, IN, VA<br>Tonsil: N, IN, VA<br>Lymph node: N, IN, VA<br>Adrenal gland: N, IN, VA<br>Mesenteric ganglia: N, IN, VA<br>Trigeminal ganglion: N, IN, VA<br>Cerebral cortex: N, IN, I, VA
Narita *et al.* 1984<br><br>10 domestic pigs	5 d	i.n.	Fever<br>Ataxia<br>Tremor<br>Vomiting	Small intestine: enteritis	CNS (V/A): N, IN<br>Trigeminal ganglion: N, IN<br>Myenteric plexus: N, IN<br>Submucous plexus: N, IN<br>Tonsil: N, IN<br>Lung: N, IN<br>Lymph node: N, IN<br>Adrenal gland: N, IN<br>Small intesine: I, N, IN

Reference	age	Inoculation route	Clinical signs	Macroscopy	Pathohistology
Baskerville *et al.* 1973<br><br>Domestic pigs	4 w	i.n.	N/A	Lung: consolidation, E<br>Lymph nodes: enlarged	Lung: N, I, IN, E
Sabo *et al*. 1968<br><br>Domestic pigs	7 d	p.o.	Non-specific and neurological signs	N/A	Tonsil: N, I, IN, VA<br>Lymph node: N, I, IN, VA<br>Lymphocytes: VA<br>Nose: I, VA<br>Cerebral/cerebellar meninges: I<br>Cerebral cortex: E, N, I, VA<br>Olfactory bulb: VA<br>Thalamus: I, VA<br>Striatum: I; VA<br>Brainstem: I, VA<br>Trigeminal ganglion: VA
Rajcáni *et al.* 1969<br><br>10 domestic pigs	7 d	s.c.	Paresis<br>Ataxia<br>Paralysis<br>Seizures	N/A	Muscle: I, E, N, VA<br>Lumbosacral spinal cord: demyelination, meningeal infiltration, VA<br>Cervical spinal cord: N, I<br>Spinal ganglia: VA<br>Brainstem: N, I<br>Cerebral meningeal infiltration<br>Lymph node: I, VA<br>Skin: VA<br>Mononuclear cells: VA

Reference	age	Inoculation route	Clinical signs	Macroscopy	Pathohistology
Corner, 1965<br><br>29 domestic pigs	1) 3 w<br>2) 1 d<br>3) 1 w<br>4) 3 d	1) i.m.<br>2-4) i.n.	1) Fever<br>Paralysis<br>Depression<br>Dyspnea<br>Dog-siiting recumbency<br>Neurological deficits<br>2) Dullness<br>Fever<br>3) Fever<br>Dullness<br>4) Fever	1) Heart: Pericardial effusion, pericarditis<br>Lung: H<br>Spleen: white foci<br>Liver: white foci<br>Testicle: white foci<br>Kidney: white foci<br>Adrenals: enlarged, nodular<br>Brain: cerebellar coning, subdural hemorrhage, hemorrhages in olfactory bulb<br>2) Lung: consolidation, E, H<br>Heart: Pericardial effusion, pericarditis<br>Kidney: H<br>3-4) Nasal discharge<br>Tonsils/tongue/nasopharynx: erosions, N<br>Spleen: enlarged<br>Heart: Pericardial effusion, pericarditis	1) Muscle: N, I<br>Sciatic nerve: I<br>Spinal ganglia: D<br>Lumbar spinal cord: D, IN, I<br>Adrenal gland: N, IN<br>Liver: N<br>Spleen: N<br>Lung: E, N, IN<br>Kidney: N<br>Heart: I<br>Nasal turbinates: N, IN<br>Testicle: N<br>Lymph node: I<br>2) Brainstem: I, IN<br>Nose: N<br>Spleen: N<br>Liver: N<br>Kidney: N<br>Adrenals: N<br>Lymph node: N<br>Lung: N<br>3-4) see 1 and 2; more CNS lesions
McFerran and Dow, 1965<br><br>40 domestic pigs	1) 8 w<br>2) 7 w	1-2) i.n.	1-2) Fever<br>Anorexia<br>Tremor<br>Ataxia<br>Nystagmus<br>Convulsions	N/A	1-2) Cerebrum: I<br>Olfactory bulb: I, IN<br>Cerebellum: I, IN<br>Brainstem: I; IN<br>Cervical/thoracic spinal cord: I

Cattle, natural infection

Reference	Clinical signs	Macroscopy	Pathohistology
Power *et al.* 1990<br><br>5y old cow	Incoordination<br>Circling<br>Salivation<br>Grunting sounds<br>Excoriation ear skin<br>Coma	Superficial skin damage	Brain (not specified): C, I, H +<br>Cerebrum: IN<br>Midbrain: G<br>Medulla: G<br>Cervical cord: G
Cheng *et al.* 2019<br><br>9 cattle	Neurological impairment<br>Salivation<br>Banging their heads against the wall	Meninges: C<br>Lung: consolidation	Cerebrum: I, N, G, VA +++
Kirkbride *et al.* 1992<br><br>5-month fetus	Abortion	none	Lung: I<br>Kidney: D of tubular epithelium
Dow and McFerran, 1962<br><br>6 cattle	Pruritus hind-quarters and perineum	N/A	Lumbar/sacral DRG: I, IN, N<br>Sacral/lumbar/thoracic/cervical spinal cord: I, N, IN, gliosis

Cattle, experimental infection

Reference	Inoculation route	Clinical signs	Macroscopy	Pathohistology
Crandell *et al.* 1982<br><br>3 calves	i.n.	Fever<br>Self-mutilation	Skin laceration/abrasion<br>Meninges: C<br>Increased CSF<br>Fibrous pleuritis	Cerebrum: I, N, G (+)<br>Cerebellum: I, N, G (+)<br>Midbrain: I, N, G (+++)<br>Brainstem: I, N, G (+++)<br>Trigeminal ganglia: I, N, G (+++)<br>Cervical spinal cord: I, N, G (+)<br>Lymph node: N<br>Tonsils: N<br>Parotid gland: N<br>Heart: I

Reference	Inoculation route	Clinical signs	Macroscopy	Pathohistology
Dow and McFerran 1966<br><br>8 calves	1) i.m. foreleg<br>2) i.v.	1) Wandering<br>Fever<br>Pruritus<br>Stamping the foreleg<br>Stumbling<br>Radial paralysis<br>Arching of the lower spine<br>Abdominal straining<br>Recumbency<br>2) Hyperexcitability<br>Muscle tremor<br>Neurological impairment	1) Skin excoriation<br>Depilation due to licking<br>Excessive cerebrospinal fluid<br>Meninges of cervical/thoracic spinal cord: C<br>2) Subcutaneous bruising<br>Excessive CSF	1) Cervical/thoracic/lumbar spinal cord: C, E, N, IN, I<br>Brachial plexus: I<br>DRG: I, N, IN<br>Brainstem: N, gliosis<br>Mesencephalon: N, G<br>2) Olfactory bulb: I, IN, N, G (+++)<br>Cerebrum: I, IN, N, G (+++)<br>Diencephalon: I, N, G (+)<br>Mesencephalon: I, N, G (+)<br>Brainstem: I, N, G (+)<br>Cerebellum: N, I (+)<br>Thoracic/lumbar spinal cord: I, N, IN, gliosis<br>DRG: N, IN<br>Adrenals: N, IN, I<br>Autonomic ganglia: N, IN
McCracken *et al.* 1973<br><br>24 calves	s.c.	N/A	N/A	Fasciae: VA<br>Spinal ganglion and lower thoracic segment: N, IN, VA

**Reference**	**Inoculation route**	**Clinical signs**	**Macroscopy**	**Pathohistology**
Dow and McFerran, 1962<br><br>10 calves	1) i.d. hip<br>2) s.c. hip<br>3) s.c. shoulder<br>4) s.c. cheek<br>5) i.n.	1 and 2)<br>Fever<br>Pruritus<br>Muscle tremor<br>Self-mutilation<br>Recumbency<br>Neurologic impairment<br>Dyspnea<br>Coma<br>3) Fever<br>Pruritus<br>Self-mutilation<br>Muscle tremor<br>Neurological impairment<br>Nystagmus<br>Coma<br>4) Pruritus<br>Self-mutilation<br>Neurological impairment<br>Coma<br>5) Neurological impairment<br>Muscle spasms<br>Nystagmus<br>Recumbency<br>Coma	1 and 2)<br>Skin excoriation<br>Lymph nodes: enlarged<br>Increased CSF<br>Meninges: C<br>Lung: E<br>Thymus: H<br>Heart: H<br>3) Skin excoriation<br>Increased CSF<br>Meninges: C<br>4) Skin edema and hemorrhages<br>Increased CSF<br>Meninges: C<br>5) Increased CSF<br>Meninges: C	1 and 2)<br>Muscle: I, N<br>Dermis: I, N<br>Lumbar/sacral DRG: I, IN, N<br>Sacral/lumbar/thoracic Spinal cord: I, N, IN, G<br>3) Dermis: I, N<br>Lumbar/sacral DRG: I, IN, N<br>Cervical/Thoracic Spinal cord: I, N<br>Brainstem: I, N, IN<br>Cerebellum: I, N, G<br>Mesencephalon: N, G<br>4) Dermis: I, N<br>TG; I, N<br>Brainstem: N, I, G,<br>Cerebellum: N, I<br>Cervical spinal cord: G, N<br>Lymph nodes: C<br>5) Lymph nodes: C<br>Lung: E<br>Olfactory bulb: N, I, IN, demyelination, gliosis (+++)<br>Cerebral cortex: N, IN, G (+++)<br>Cerebral vessels: I,N<br>Brainstem: N, I

Sheep and goat, natural infection

Reference	Clinical signs	Gross pathology	Pathohistology
Henderson *et al.* 1995<br><br>29 sheep	Depression<br>Anorexia<br>Fever<br>Pruritus<br>Neurological impairment	Skin excoriation/depilation<br>Lung: E, C<br>Heart: H	Lung: C, E<br>Brain: none<br>Spinal cord: none
Olander *et al.* 1966<br><br>1 sheep	N/A	N/A	Brainstem: I, N, G<br>Cerebellum: I, N, G<br>Midbrain: I, N, G
Baker *et al.* 1982<br><br>1 goat	Pruritus<br>Facial swelling<br>Recumbany<br>Dyspnea<br>Coma	Skin lacerations	Brain (not specified): I, IN
Herweijer and DeJonge, 1977<br><br>5 goats	Agitation<br>Restlessness<br>Vocalization<br>Sweating<br>Muscle spasms<br>Neurological impairment	N/A	N/A

Sheep and goat, experimental infection

Reference	Inoculation route	Clinical signs	Macroscopy	Pathohistology
Schmidt *et al.* 1987<br><br>24 sheep	i.t.	Fever<br>Depression<br>Salivation<br>Fasciculation<br>Dyspnea<br>Tympany<br>Self-mutilation	Skin lacerations<br>Heart: H<br>Lymph nodes: enlarged<br>Dilated oesophagus<br>Cervicothoracic ganglia: H	Heart: I<br>Lung: VA<br>Lymph nodes: N, VA<br>Spleen: N<br>Parabronchial ganglia: IN<br>Trigeminal ganglia: IN, I, VA<br>Cranial cervical ganglia: I, H, N, IN, VA<br>Cervicothoracic ganglia: I, H, N, IN, VA<br>Mesencephalon: IN, VA<br>Metencephalon: IN<br>Medulla oblongata: IN, I, VA<br>Spinal cord: VA
Mocsari *et al.* 1987<br><br>5 lambs	i.n.	Coughing<br>Nasal discharge<br>Dyspnea<br>Pruritus<br>Abnormal position of head/neck<br>Hyperexcitability<br>Convulsions	Skin lacerations<br>Nasal mucose/pharynx: C<br>Lung: E	Brainstem: I, G<br>Cervical spinal cord: I<br>Nose: D, I<br>Pharynx: D, I, IN<br>Lung: E, H, I, D

Dogs and cats, natural infection

Reference	Clinical signs	Macroscopy	Pathohistology
Engelhardt *et al.* 2019<br><br>1 dog	Pruritus<br>Neurological deficits	N/A	Brainstem: I, VA (+++)
Pedersen *et al.* 2018<br><br>dogs	Pruritus<br>Head edema<br>Vomiting<br>Self-mutilation<br>Vocalization	N/A	Brainstem: I, IN, G (++)<br>Liver: I<br>Intestine: I
Serena *et al.* 2018<br><br>1 dog	Neurological deficits	N/A	Cerebrum: I, G (+)
Zhang *et al.* 2015<br><br>13 dogs	Pruritus<br>Neurological deficits<br>Salivation<br>Vomiting<br>Dyspnea	Heart: H<br>Stomach: H<br>Kidney: H<br>Thymus: H<br>Lung: C	Brainstem: I, VA (+++)<br>Lung: H<br>Liver: N<br>Thymus: H, LD<br>Kidney: I
Schöniger *et al.* 2012<br><br>1 dog	Neurological deficits<br>Muscle spasms<br>Hypothermia<br>Dyspnea<br>Vomitus<br>Diarrhea	Heart: H<br>Lung: emphysema, E	Brainstem: I, IN, G, VA (++)<br>Trigeminal ganglion: VA<br>Myenteric plexus: VA<br>Stomach (fibrocytes, glands): VA<br>Heart (cardiomyocytes): VA
Campbell *et al.* 2011<br><br>3 dogs	Pruritus<br>Self-mutilation<br>Vomiting<br>Diarrhea<br>Fever<br>Muscle spasm	Heart: H<br>Skin abrasions<br>Dark intestinal content<br>Lymph nodes: enlarged	Trigeminal ganglion: I, IN, VA (+++)<br>Brainstem: I (+)<br>Skin: I (+++)<br>Lung: E<br>Muscles: D

Reference	Clinical signs	Macroscopy	Pathohistology
Quiroga *et al.* 1998<br><br>7 dogs	Neurological deficits<br>Salivation<br>Pruritus<br>Vomiting<br>Lethargy<br>Coma	Enteritis<br>Thoracic/pericardial effusion<br>Lung: C<br>Balck stomach content<br>Tonsils: enlarged<br>Meninges: C	Brainstem: I, IN, G, VA (+++)<br>Diencephalon: I (+), VA<br>Cervical spinal cord: I, VA (+)
Hara *et al.* 1991<br><br>1 cat	Vomiting<br>Salivation,<br>neurologic deficits,<br>Pruritus<br>Lethargy	N/A	Brainstem: I, IN, H<br>Cervical/thoracic/lumbar spinal cord: I, IN<br>Abdominal plexus/ganglia (N/A): I, IN<br>Lumbar ganglia: VA<br>Intestinal tract: VA<br>Liver: N<br>Tonsil/pharyngeal Lnn: N
Monroe *et al.* 1989<br><br>25 dogs	Salivation<br>Anorexia<br>Neurologic deficits<br>Dyspnea<br>Vocalization<br>Pruritus<br>Vomiting<br>Agressiveness<br>Dysphagia	N/A	N/A
Kelly and Ratcliffe, 1983<br><br>1 dog	Pruritus<br>Vomiting<br>Fever	Stomach/intestine: H<br>Black stomach content	Plexus myentericus: I, N, IN<br>Encephalon (N/A): I<br>Adrenal medulla: I
Shell and Grandell, 1981<br><br>1 dog	Salivation<br>Lethargy<br>Vomiting	none	Brainstem: I, IN, G (+++)<br>Lung: C<br>Liver: D
Gore *et al.* 1977<br><br>11 dogs	Dyspnea<br>Anorexia<br>Vocalization<br>Salivation<br>Prurritus<br>Vomiting<br>Diarrhea	Head edema<br>Esophageal/gastric ulceration<br>Black stomach content<br>Enteritis<br>Liver: D<br>Lung: C	Encephalon (N/A): I (+)<br>Cerebellum: N<br>Plexus myentericus: I, N, IN (+++)<br>Adrenal medulla: I, N, IN (+++)

Reference	Clinical signs	Macroscopy	Pathohistology
Horvath and Papp, 1967<br><br>58 cats	Salivation<br>Vomiting<br>Lethargy<br>Aggressiveness<br>Pruritus<br>Skin abrasions<br>Anisocoria	N/A	N/A
Olander *et al.* 1966<br><br>2 dogs	N/A	N/A	Brainstem: I, N, G<br>Midbrain: I, N, G<br>Cerebellum: I, N, G

Dogs and cats, experimental infection

Reference	Inoculation route	Clinical signs	Macroscopy	Pathohistology
Yin *et al.* 2020<br><br>4 dogs	i.m	Anorexia<br>Neurological deficits<br>Muscle spasms	N/A	Encephalon (N/A): I, N<br>Lung: I, N<br>Stomach: N<br>Liver: C, H
Zhang *et al.* 2015<br><br>5 dogs	s.c.	Pruritus<br>Self-mutilation<br>Salivation<br>Anorexia<br>Vocalization<br>Depression<br>Dyspnea	Heart: H<br>Lung: H<br>Gut reddening<br>Head edema<br>Ascites	Brainstem: VA<br>Cervical spinal cord: VA<br>Stellate ganglion: I (+++), N, IN, G, VA<br>Celiac ganglion: I, VA<br>Caudal mesenteric ganglion: VA<br>Heart: H, N<br>Lung: C<br>Small intestine: C, LD<br>Large intestine: H<br>Stomach: N<br>Thymus: H, LD<br>Lymphnodes: H, LD<br>Adrenal gland: H

Reference	Inoculation route	Clinical signs	Macroscopy	Pathohistology
Olson and Miller, 1986<br><br>16 dogs	i.v.<br>i.m.<br>s.c.<br>o.n.	N/A	Heart: H<br>Lung: C, E	Heart: I, IN, H<br>Stellate ganglion: I, IN, N
Hagemoser *et al.* 1980<br><br>29 cats	p.o.	N/A	None	Lung: I<br>Tonsil: I<br>Brainstem: I, G (++)<br>Midbrain: I, G (+)<br>Diencephalon: I, G (+)<br>Cerebellum: G<br>Spinal cord: I, G (+)<br>Trigeminal ganglion: I, G (+)
Biancifiori *et al.* 1977<br><br>10 dogs	s.c.<br>i.m.<br>fead infected meat	N/A	N/A	Brainstem: VA<br>Cervical/thoracic/lumbar spinal cord: VA<br>Pancreas: VA<br>Stomach: VA<br>Parotid gland: VA

Wild carnivores, natural infection

Reference	Clinical signs	Gross pathology	Pathohistology
Moreno *et al.* 2020<br><br>1 red fox	Neurologic impairment<br>Diarrhea	Hemorrhagic gastritis/enteritis	Kidney: vasculitis, tubular dilation
Liu *et al.* 2017<br><br>379 mink	Anorexia<br>Pruritus<br>Self-mutilation<br>Vomiting<br>Diarrhea<br>Dyspnea	Spleen: C<br>Intestinal erythema<br>Heart: H<br>Kidney: H<br>Lung: H, C	N/A

Reference	Clinical signs	Gross pathology	Pathohistology
Masot *et al.* 2016<br><br>1 Iberian lynx	N/A	Alopecia neck<br>Black stomach and intestinal content<br>Meninges: C	Encephalon (N/A): I, N, IN, G<br>Cerebrum/cerebellum: demyelination<br>Stomach: I<br>Small intestine: I, N
Jin *et al.* 2016<br><br>1,200 farmed foxes	Fever<br>Anorexia<br>Vomiting<br>Dyspnea<br>Pruritus<br>Vocalization	N/A	N/A
Verpoest *et al.* 2014<br><br>3 wolves	Neurological impairment	Lung: C	N/A
Caruso *et al.* 2014<br><br>1 red fox	Neurologic impairment<br>Pruritus	Head edema<br>Skin abrasions	N/A
Raymond *et al.* 1997<br><br>3 coyotes	Anorexia<br>Vocalization	Skin abrasions<br>Neck edema and hemorrhages<br>Black intestinal content	Encephalon (N/A): I, H, N, IN, G<br>Skin: I
Zanin *et al.* 1997<br><br>5 brown bears	Depression<br>Anorexia<br>Salivation<br>Pruritus<br>Self-mutilation<br>Dyspnea<br>Paralysis	Skin abrasions	N/A
Glass *et al.* 1994<br><br>1 Florida panther	N/A	Peritoneal and thoracic effusion<br>Dark red content in eosophagus, stomach and intestine<br>Intestinal erosions<br>Meninges: C	N/A

Reference	Clinical signs	Gross pathology	Pathohistology
Schultze *et al.* 1986<br><br>1 black bear	Lethargy<br>Depression<br>Anorexia<br>Neurologic impairment<br>Vocalization	Lung: C, E<br>Aspiration of ingesta<br>Ascites<br>Volvulus small intestine<br>Mesenteric lymph nodes: C	Lung: E<br>Spleen: LD<br>Liver: C, N, IN<br>Adrenal gland: H, N, IN<br>Mesenteric lymph nodes: C, E
Bitsch *et al.* 1971<br><br>12 red foxes	Apathy<br>Salivation<br>Neurologic impairment<br>Pruritus	Alopecia head, tail, limbs<br>Skin abrasions head, limbs	N/A
Kirkpatrick *et al.* 1980<br><br>6 racoons	N/A	N/A	N/A

Wild carnivores, experimental infection

Reference	Inoculation route	Clinical signs	Macroscopy	Pathohistology
Quiroga et al. 1995<br><br>Blue foxes	p.o.	Anorexia<br>Depression<br>Coma	none	Brainstem: I, VA, N<br>Cervical spinal cord: I, VA, N
Kirkpatrick et al. 1980<br><br>3 racoons	p.o.	Salivation<br>Neurologic impairment<br>Anorexia<br>Pruritus	Lung: C<br>Heart: H<br>Meninges: C	Tonsil: VA<br>Brainstem: VA<br>Cerebellum: VA<br>Cerebrum: VA

Horses, natural infection

Reference	Clinical signs	Macroscopy	Pathohistology
Van den Ingh *et al.* 1990<br><br>1 horse	Fever<br>Behavioral abnormalities<br>Neurological impairment<br>Blindness<br>Muscle tremor<br>Depression<br>Exhaustion	Skin lesions<br>Subcutaneous hemorrhages<br>Atlas fracture<br>Meninges: C	Cerebrum: N, I, IN, G (+++)<br>Brainstem: I, G (+)<br>Cerebellum: I, G (+)
Kimman *et al.* 1991<br><br>1 horse	Anorexia<br>Depression<br>Neurological impairment<br>Leg edema<br>Restlessness<br>Sweating<br>Muscle tremor<br>Blindness<br>Salivation	Meninges: C	Diencephalon: I, N, IN, G (+++)<br>Cerebrum (ventrolateral): I, N, IN, G (+++)

Horses, experimental infection

Reference	Infection route	Clinical signs	Macroscopy	Pathohistology
Van den Ingh *et al.* 1990<br><br>2 horses	i.n.	Fever	N/A	N/A
Kimman *et al.* 1991<br><br>2 horses	i.n.	Anxiety<br>Trembling<br>Fever<br>Anorexia<br>Depression<br>Sweating<br>Muscle tremor	Cyanotic/congested mucosae<br>Purulent nasal discharge<br>Heart: H	Olfactory bulb: N, I<br>Cerebrum: N, I<br>Diencephalon: N, I<br>Brainstem: N, I<br>Heart: N, H

Chickens, natural infection

Reference	Clinical signs	Macroscopy	Pathohistology
Kouwenhoven *et al.* 1982<br><br>> 10 000 chickens	Sluggishness<br>Lying down<br>Peracute death<br>Paralysis<br>Trembling	N/A	Cerebrum: I, gliosis, N, H

Chickens, experimental infection

Reference	Infection route	Clinical signs	Macroscopy	Pathohistology
Kouwenhoven *et al.* 1982<br><br>1-4) day old chickens<br>5) 16-days old chickens<br>6-7) one-day-old chickens	1) i.c.<br>2) i.p.<br>3) p.o.<br>4) i.m.<br>5) i.m.<br>6) i.o.<br>7) i.m.	1) Peracute death<br>Excitation<br>2) Peracute death<br>3) None<br>4) Peracute death<br>Trembling<br>5) None<br>6) None<br>7) Paralysis<br>Excitation	1) Gelatinous, yellow-orange edema from neck to beak<br>Swollen meninges<br>2) None<br>3) None<br>4) Brain: H<br>Intestine: watery content<br>5) None<br>6) None<br>7) N/A	1-2) Encephalon (not specified): I<br>3) none<br>4) N/A<br>5) N/A<br>6) N/A<br>7) Encephalon (not specified): I<br>Spinal cord: I, N, IN, N
Ramachandran and Fraser, 1971<br><br>Chickens of different age	1) i.c.<br>2) i.m.<br>3) Skin scarification<br>4) s.c.<br>5) i.n.<br>6) i.o.	1-4) Malaise<br>Anorexia<br>Drowsiness<br>Gasping<br>Tremor<br>Incoordination<br>Nasal discharge<br>2) Paresis	N/A	N/A

Laboratory animals, experimental infection

Reference	Inoculation route	Clinical signs	Macroscopy	Pathohistology
Sehl *et al.* 2020<br><br>Mice	i.n.	Pruritus<br>Depression<br>Conjunctivitis<br>Self-mutilation<br>Apathy<br>Hyperactivity	Head edema<br>Skin abrasions	Nose: VA<br>Nasopharynx: VA<br>Salivary glands: VA<br>Trigeminal ganglion: VA<br>Brainstem: VA<br>Superior cervical ganglion: VA, I<br>Pterygopalatine ganglion: VA
Laval *et al.* 2018<br><br>Mice	Foot pad	Pruritus	N/A	Dorsal root ganglia: I<br>Skin: I
Klopfleisch *et al*. 2004, 2006<br><br>Mice	i.n.	Anorexia<br>Apathy<br>Depression<br>Pruritus<br>Self-mutilation<br>Hyperactivity<br>Neurologic deficits<br>Dyspnea	Head edema<br>Skin abrasions	Nose: VA<br>Trigeminal ganglion: VA<br>Brainstem: VA<br>Midbrain: VA<br>Superior cervical ganglion: VA<br>Pterygopalatine ganglion: VA
Brittle *et al*. 2004<br><br>Mice	s.c. (flank)	Pruritus<br>Self-mutilation<br>Neurologic deficits<br>Depression<br>Lethargy	Skin abrasions	Brainstem: VA<br>Cerebellum: VA
Sabini *et al.* 2000<br><br>Rabbits	s.c.	Neurologic deficits<br>Hyperactivity<br>Pruritus<br>Self-mutilation<br>Rough fur	Skin abrasions	N/A
Rassnick *et al.* 1998<br><br>Rats	i.o.	N/A	N/A	Cerebrum: VA<br>Midbrain: VA<br>Cranial nerves III, IV, VI: VA

Reference	Inoculation route	Clinical signs	Macroscopy	Pathohistology
Babic *et al.* 1994<br><br>Mice	i.n.	Pruritus<br>Self-mutilation<br>Hyperactivity	N/A	Nose: VA<br>Vomeronasal organ: VA<br>Blood vessels: VA<br>Brainstem: VA<br>Trigeminal ganglion: VA<br>Superior cervical ganglion: VA
Card *et al.* 1991<br><br>Rats	i.o.	N/A	N/A	Retina: VA<br>Cerebrum: VA<br>Midbrain: VA
Schijns *et al.* 1989<br><br>Rats	i.p.	N/A	N/A	Peritoneum: VA<br>Myenteric plexus: VA<br>Submucous plexus: VA<br>Abdominal sympathetic ganglia: VA<br>Mesenteric ganglia: VA<br>Dorsal root ganglia: VA<br>Spinal cord: VA<br>Adrenal gland: VA
Ashworth *et al.* 1980<br><br>Guinea pig	i.n.	Pruritus<br>Self-mutilation<br>Fever<br>Neurologic impairment	N/A	Cerebrum (olfactory bulb, hippocampus, cerebral cortex): I, IN, N, gliosis<br>Midbrain: I, N, IN, gliosis<br>Brainstem: I, N, IN, gliosis
Field and Hill, 1974<br><br>Mice	Foot pad	Neurologic deficits<br>Pruritus<br>Self-mutilation	N/A	N/A
McFerran and Dow, 1970<br><br>Rats	1) p.o.<br>2) s.c. shoulder<br>3) i.n.	1) Pruritus<br>2 and 3) Depression<br>Ruffled fur<br>Facial irritation	Skin abrasions	1-3) Vessels: endothelial hyperplasia<br>Encephalon (N/A): I, N, IN
Fraser and Ramachandran, 1969<br><br>Mice	i.n.<br>i.o.<br>i.c.<br>i.v.<br>i.m.<br>i.p.	Recumbancy<br>Coma<br>Pruritus<br>Self-mutilation	N/A	N/A

Reference	Inoculation route	Clinical signs	Macroscopy	Pathohistology
Fraser and Ramachandran, 1969<br><br>Rats	i.n.<br>i.o.<br>i.c.<br>i.v.<br>i.m.<br>i.p.	Similar to mice<br>Diarrhea<br>Orchitis	N/A	N/A
Olander *et al.* 1966<br><br>Rabbits	s.c.	N/A	Skin abrasions	Skin: I, H, E<br>Lung: C, E<br>Liver: C, N, I
Dempsher et al., 1955<br><br>Rats	i.o.	Pruritus	N/A	Superior cervical ganglia: I, N, IN
Hurst, 1936<br><br>Rhesus macaques	1) i.c.<br>2) intrasciatic	1-2)<br>Fever<br>Apathy<br>Salivation<br>Neurological deficits	None	1) Cerebrum: N, I<br>Basal ganglia: I, N<br>Brainstem: I, N<br>Cerebellum: none<br>2) Spinal ganglia: N, I<br>Lumbar, cervical spinal cord: N, I<br>Brainstem: I, N<br>Cerebrum: I, N

Human, natural infection

Reference	History	Clinical signs	Clinical findings (MRI, NGS)
Yang *et al.* 2019<br><br>1 patient	Veterinarian, hand injury from swine necropsy	Fever<br>Headache<br>Seizures	Lesions in basal ganglia, occipital lobe, limbic lobe, thalamus
Yang *et al.* 2019<br><br>5 patients	1) Pig slaughterer, finger injury<br>2) Pork cutter<br>3) Pig handler, hand injury<br>4) Pork cutter, hand injury<br>5) Pork cutter	1) Headache<br>Visual impairment<br>Altered mental status<br>Convulsions<br>Incontinence<br>2) Fever<br>Memory loss<br>Convulsions<br>Respiratory failure<br>3) Fever<br>Altered mental status<br>Tremor<br>Repiratory failure<br>4) Fever<br>Convulsions<br>Respiratory failure<br>5) Fever<br>Convulsions<br>Altered mental status<br>Repiratory failure	1) Lesions in frontal lobe, insular cortex<br><br>PrV detection in CSF via NGS<br><br>2) Lesions in the temporal lobes, insular cortex<br><br>PrV detection in CSF via NGS<br><br>3) Lesions in the temporal lobes, insular cortex<br>Retinal arterial stiffness<br>Optic nerve atrophy<br><br>PrV detection in CSF and vitreous humor via NGS<br><br>4) Lesions in temporal lobes, insular cortex<br><br>PrV detection in CSF via NGS<br><br>5) Lesions in temporal lobes, insular cortex, caudate nucleus, cingulate gyrus, thalamus, basal ganglia<br>Optic nerve atrophy<br><br>PrV detection in CSF via NGS

Reference	History	Clinical signs	Clinical findings
Zhao *et al.* 2018<br><br>4 patients	Exposure to raw pork	Fever<br>Convulsions<br>Loss of consciousness<br>Respiratory failure	Retinitis<br>Lesions in limbic system, basal ganglia, midbrain<br><br>PrV detection via NGS<br>Detection of PrV antibodies
Ai *et al.* 2017<br><br>1 patient	Exposure to sewage on a hog farm	Fever<br>Headache<br>Visual impairment	Endophthalmitis<br><br>PrV detection in vitreous humor via NGS<br>Detection of PrV antibodies
Anusz *et al.* 1992<br><br>6 patients	Pig and cattle farm workers	Pruritus of palms, lower and upper arms, shoulder, back	N/A
Mravak *et al.* 1987<br><br>3 patients	1) Cut his thumb while washing dishes of the cat<br><br>2 and 3) had close contact to cats and other animals	1) Weakness<br>Fever<br>Sweating<br>Dysphagia<br>Dysgeusia<br>Pain in the tongue<br>Hypersalivation<br>Loss of appetite/weight<br>Headache<br>Tinnitus<br>Pain of muscles/joints<br>Paraesthesia<br>2) Tiredness<br>Fever<br>Sweating<br>Diarrhea<br>Enlarged lymph nodes<br>Dysphagia<br>3) Tiredness<br>Fever<br>Sweating<br>Diarrhea<br>Enlarged lymph nodes<br>Dysphagia	1-3) Detection of PrV antibodies

Reference	History	Clinical signs	Clinical findings
Hussel *et al.* 1963<br><br>4 patients	1) Animal handler, exposure to infected dog<br>2) Animal handler, exposure to infected dog<br>3) Nightwatchman, exposure to infected dog<br>4) Veterinarian, exposure to infected dog	Throat pain<br>Weakness	none
Schükrü-Aksel and Tuncman, 1940<br><br>2 patients	1) Lab worker, self-injury during necropsy of a PrV-infected dog<br>2) Lab worker, self-injury while handling the PrV-infected dog	Pruritus<br>Erythema<br>Pain<br>Aphthous stomatitis	Diagnosis of AD in a rabbit after inoculation of patient's serum
von Ratz *et al.* 1914<br><br>2 patients	1) Lab technician, hand injury, exposed to PrV-Infected laboratory cat<br>2) Lab technician, hand injury, exposed to brain emulsion from the PrV-infected laboratory cat	Pruritus/swelling of the wound	none

Human, experimental infection

Reference	Inoculation route	Clinical signs
Jentzsch and Apostoloff, 1970<br><br>Self-inoculation	s.c.	Erythema at inoculation site
